# Coconut Oil Nanoemulsion Loaded with a Statin Hypolipidemic Drug for Management of Burns: Formulation and In Vivo Evaluation

**DOI:** 10.3390/pharmaceutics12111061

**Published:** 2020-11-07

**Authors:** Khaled M. Hosny, Nabil A. Alhakamy, Amal M. Sindi, Rasha A. Khallaf

**Affiliations:** 1 Department of Pharmaceutics, Faculty of Pharmacy, King Abdulaziz University, Jeddah 21589, Saudi Arabia; nalhakamy@kau.edu.sa; 2 Center of Excellence for Drug Research and Pharmaceutical Industries, King Abdulaziz University, Jeddah 21589, Saudi Arabia; 3 Department of Pharmaceutics and Industrial Pharmacy, Faculty of Pharmacy, Beni-Suef University, Beni-Suef 62511, Egypt; rasha.mahmoud@pharm.bsu.edu.eg; 4 Oral Diagnostic Science Department, Faculty of Dentistry, King Abdulaziz University, Jeddah 21589, Saudi Arabia; amsindi@kau.edu.sa

**Keywords:** nanoemulsion, burns, wound healing, coconut oil, simvastatin, experimental design

## Abstract

Burn wound healing is a complex process that involves the repair of injured tissues and the control of infection to diminish the scar formation, pain, and discomfort associated with such injuries. The aim of this research was to formulate and optimize a self-nanoemulsion drug delivery system based on the use of coconut oil and loaded with simvastatin. Coconut oil possesses antiinflammatory and antibacterial activity, and simvastatin has interesting properties for promoting the wound-healing process because it increases the production of the vascular endothelial growth factor at the site of injury. The Box–Behnken design was employed for the optimization of the coconut oil–simvastatin self-nanoemulsion drug delivery system. The prepared formulations were characterized according to globular size and their activity in the healing of burn wounds by assessing the mean wound diameter and level of interlukin-6 in experimental animals. Additionally, the antimicrobial activity of the prepared formulations was assessed. The nanoemulsion was considered adequately formed when it had droplets of between 65 and 195 nm. The statistical design proved the important synergistic effect of coconut oil and simvastatin for burn wound management in their synergistic potentiation of wound closure and their anti-inflammatory and antimicrobial effects. The optimum formulation achieved up to a 5.3-fold decrease in the mean burn wound diameter, a 4.25-fold decrease in interleukin-6 levels, and a 6-fold increase in the inhibition zone against *Staphylococcus aureus* when compared with different control formulations. Therefore, the designed nanoemulsions containing a combination of coconut oil and simvastatin could be considered promising platforms for the treatment of chronic and burn wounds.

## 1. Introduction

Severe burns and chronic wounds are among the common causes of extensive morbidity and death. Wound healing is a complex process involving the repair of injured tissues and control of infections to diminish the scar formation, pain, and discomfort associated with such injuries [1]. Wound healing is a dynamic, complex physiological process that involves a variety of cells, which contribute to a series of interacting stages such as inflammation, revascularization, epithelialization, maturation, and subsequent remodeling of newly developed tissues [2].

Burns are skin wounds that can affect significant portions of the human body. Controlling scar formation and microbial infection are the main concerns in the healing of such wounds [3]. Many researchers have investigated techniques to enhance the antimicrobial activity of topically applied agents that target and repair injured tissues. Recently, the emergence of multiresistant bacterial strains presented a serious challenge to the development of an effective drug delivery system for patients suffering from infected lesions [4]. Naturally occurring materials such as honey, aloe, and plant oils have been presented as potential antibacterial agents [5]. Some plant oils, in particular, exhibit antibacterial activity against multiresistant bacteria, owing to a broad spectrum of biologic and antimicrobial effects [6].

Virgin coconut oil (CnO) is extracted from *Cocos nucifera* Linn (Arecaceae), which grows in tropical zones [7]. The oil is composed predominantly of medium-chain fatty acids, especially lauric acid, along with other fatty acids, such as oleic and linoleic acids. CnO is known to be easily absorbed, with a high potential for accelerating cell metabolism, moistening wounds, and decreasing inflammatory signs [8]. Coconut oil hydrolysis usually produces monoglycerides, diglycerides, and free fatty acids (FFAs). A combination of monoglycerides and FFAs and, principally, lauric acid and monolaurin is known to have antibacterial and antifungal effects, and this makes coconut oil a good candidate for use in wound-healing formulations [9].

Statins are a class of drugs that is well known for its antihypercholesterolemic effects. Statins exert their actions through competitively inhibiting 3-hydroxy-3-methylglutaryl coenzyme A (HMG-CoA) reductase and, hence, lowering cholesterol biosynthesis [10]. Recently, several pleiotropic effects of statins have been described, including anti-inflammatory actions [11], the enhancement of microvascular and endothelial functions [12], the modulation of endothelial nitric oxide synthase activity [13], ischemia/reperfusion [14], and improved outcomes for patients with sepsis [15]. Simvastatin (SMV) is one statin that has interesting activity in promoting the wound-healing process [16]. Its mechanism of action includes increasing the production of vascular endothelial growth factor at the site of injury, which is a crucial step in the synthesis of new blood vessels [17]. SMV is also thought to decrease synthesis and the release of mevalonate and farnesyl pyrophosphate, leading to the enhancement of the epithelialization and revitalization of the epidermis. Decreasing the farnesyl pyrophosphate level, increased the in vitro migration of keratinocytes and the tissue repair during a previously reported ex vivo human culture wound-healing experiment [18]. Furthermore, SMV was proved to have good antimicrobial activity against gram-positive bacteria, and, hence, it could be considered a good solution to serious problems such as burns and chronic wounds [19].

A nanoemulsion is a drug delivery system formed from small droplets of 20–200 nm enclosed by a surfactant layer [20]. It offers several advantages over conventional emulsions, such as (1) a good ability to target drugs to action sites due to a very large interfacial area, (2) the protection of drugs against hydrolysis and enzymatic degradation, (3) the improvement of drug loading, (4) the increase of drug solubility and bioavailability, (5) the reduction of intrapatient variation, and (6) the achievement of a controlled drug release [21]. A nanoemulsion is frequently formulated using low concentrations of a surfactant [22], so choosing the type of surfactant is a critical step in formulating required characteristics such as long-term stability. Such stability could be achieved by generating a surfactant layer that surrounds droplets of the inner phase, which would strengthen the interfacial film and confirm nanoemulsion stability [23].

A design of experiments approach was recently used in the formulation and optimization of several drug delivery systems to study the effect of variable factors on certain measured responses [24]. That approach has many good features, such as (1) using fewer experiments to reach the optimum formulation, (2) making it easier to allocate and trace problems, (3) detecting drug/excipient interactions, and (4) predicting the formulation performance and the ability to improve it [25]. Optimization methodology usually gives the best composition of the formulation and, hence, is considered a cost-effective method [26]. The aim of the current investigation was to formulate a coconut oil-based nanoemulsion loaded with SMV and deliver it by a topical route to examine its effect in promoting and enhancing the wound-healing process.

## 2. Materials and Methods

### 2.1. Materials

SMV was acquired as a generous gift from the Saudi Arabian Japanese (SAJA) Pharmaceutical Company Limited (Jeddah, Saudi Arabia). Coconut oil was purchased from Avanti Polar Lipids (Alabaster, AL, USA). Cremophor EL was purchased from Sigma-Aldrich Co. (St Louis, MO, USA). Lauroglycol™ FCC was a gift from Gattefosse (Saint-Priest, France). High-performance liquid chromatography–grade methanol and acetonitrile were obtained from Merck (Darmstadt, Germany). Chloroform, absolute ethanol, phosphate buffer pH 7.4, and 0.9% normal saline were purchased from Fisher Scientific UK (Loughborough, Leicestershire, UK). Water was obtained from a Milli-Q water purification system (Millipore, Bedford, MA, USA). All other reagents and chemicals were of analytical grade.

### 2.2. Methods

#### 2.2.1. Experimental Design and Optimization of Self-Nanoemulsion Formulations

A response surface Box–Behnken design was applied to investigate the effects of independent variables on the in vitro characteristics and in vivo efficacy parameters for the prepared self-nanoemulsion drug delivery system (SNEDDS) formulations using Design-Expert^®^ software v. 12.0.6.0 (Stat-Ease, Inc., Minneapolis, MN, USA). The independent variables were (1) the amount of coconut oil (CnO) in milligrams, (2) the amount of SMV in milligrams, and (3) the Smix ratio (ratio of surfactant (Cremophor EL) to the cosurfactant (Lauroglycol) in the prepared nanoemulsion). The investigated dependent responses were the globule size of the prepared SNEDDS (Y_1_), mean burn wound diameter (Y_2_), interleukin-6 value (Y_3_), and inhibition zone against *Staphylococcus aureus* (Y_4_). The independent factors and determined responses are shown in Table 1.

#### 2.2.2. Self-Nanoemulsion Preparation

The technique involved the preparation of a loaded nanoemulsion formulation applied in two stages. The first stage included the formation of the plain SNEDDS, in which a coconut oil concentration of 12.5%, 17.5%, or 22.5% (according to the design) was mixed with 87.5%, 82.5%, or 77.5%, respectively, of the surfactant and cosurfactant mixture (Smix). The surfactant and cosurfactant were mixed in three different ratios (1:1, 2:1, and 3:1), according to the design. In the second stage, the solid ingredient, SMV, was mixed with the plain SNEDDS with the aid of sonication in concentrations of 5, 10, or 20 mg/g of the plain self-nanoemulsion according to the design, as is shown in Table 2.

#### 2.2.3. Determination of Globule Size

The globule size of the formulated SMV-CnO SNEDDS was measured by diluting 200 μL of the formulation with 800 μL of purified water in a volumetric flask. Following vigorous mixing of the formulation, 200 μL of the dispersed sample was used to determine the globule size by a Microtrac^®^ zeta track particle size analyzer (Microtrac, Inc., Montgomeryville, PA, USA). Samples were diluted with purified water with a ratio of 1:10 and subjected to analysis in the used instrument using the dynamic light scattering technique.

#### 2.2.4. Animal Handling and Care

Animal handling and care were performed according to the guidelines of the Animal Ethics Committee, University of Jeddah, Saudi Arabia. Researchers complied with guidelines set forth in the Declaration of Helsinki and its Guiding Principles in the Care and Use of Animals (NIH Publication No. 85-23, 1985 revision), following ethical approval of the protocol before experimentation (Approval No.17-7-20) at 26th July 2020. The experimental rats were purchased and kept in laboratory cages with free access to food and water. Sufficient care of the animals was taken to minimize suffering. Acclimatization of the animals was done for a minimum of 14 days before experimentation under standard conditions of a 25 ± 1 °C temperature and a 55 ± 5% relative humidity with a 12 h light and 12 h dark cycle. The test was performed in two stages. In the first stage, 57 adult rats were divided into 19 groups, with 3 rats per group. Each group was treated with one of the formulations designed by the experimental design. The rats were anesthetized with an intraperitoneal administration of 15 mg/kg thiopental simultaneously with intramuscular administration of 25 mg/kg of ketamine in order to induce the wounds. The hair on the rat’s back was shaved, and the skin was sterilized with an alcohol swab. Then the burn wounds were introduced on the back skin by using 1.5 cm skin biopsy heated bunches. After that 1 g of the tested formulation for each group was applied topically once daily for a period of 14 days. The required dependent variables in the experimental design were determined in each group. In the second stage, the same test was repeated on a new group of 15 rats divided into 5 groups. The first group was treated with the optimum formulation suggested by the software according to the results of the first stage of the animal test. The second group was treated with topical administration of the optimum formulation prepared without incorporation of the SMV within the formula. The third group was treated with topical administration of the optimum formula prepared with the use of oleic acid instead of coconut oil. The fourth group was treated with the aqueous dispersion of SMV. The fifth group was treated with topical washing with normal saline. The test was performed for 14 days and the required measurable parameters were determined.

#### 2.2.5. Assessment of Wound Healing

##### Measurement of Burn Wound Diameter

After application of each formulation for 14 days, the rats in each group were tested by measuring the diameter of the burn wound lesion using the caliber (Mituyoto, Tokyo, Japan) to determine the second response for each group in the experimental design.

##### Interleukin-6 Measurements

For IL-6, the assay used the quantitative sandwich enzyme immunoassay technique (R&D Systems, Inc., Minneapolis, MN, USA). A monoclonal antibody specific for rat IL-6 was precoated onto a microplate. Any rat IL-6 present in the sample was bounded by an antibody. After removal of any unbounded substance, an enzyme-linked polyclonal antibody specific for rat IL-6 was added, the blue product was converted to a yellow one, and the intensity of the color was measured to determine the level of IL-6 in each of the tested samples.

#### 2.2.6. Antibacterial Evaluation

The antibacterial activity of the prepared SNEDDS was evaluated by the disc diffusion test. The Gram-positive bacterium *S. aureus*, commonly found in infected burns, was used as test bacteria. According to the method recommended by the Clinical and Laboratory Standards Institute, a suspension of *S. aureus* was made to the 0.5 McFarland turbidity standard and plated onto Mueller–Hinton agar (Difco, MI, USA). The disc specimen (with a diameter of 10 mm) was placed on the center of the agar plate and incubated at 37 °C for 24 h. If inhibitory concentrations were reached, a clear (inhibition) zone without colonies could be seen around the disc specimens. The width of the inhibition zone was measured for each tested SMV-CnO SNEDDS.

After the obtaining of the design analysis results, the optimized formula was prepared and characterized for all previously mentioned evaluation parameters as the different formulation of the design in addition to measurement of the zeta potential (ZP) and polydispersity index (PDI) in order to investigate its stability using electrophoretic mobility technique employing a Malvern Zeta sizer (Malvern instruments, Malvern, UK) at fixed angle 90° and a temperature of 25 °C. Samples were diluted with distilled (dilution ratio was 1:9 of sample: water respectively) prior to analysis; measurements were made in triplicates.

## 3. Results and Discussion

Burns are a serious problem that is very common worldwide. They might be the most life-threatening injury among all wounds [27]. Both Gram-positive and Gram-negative bacteria, such as *Pseudomonas aeruginosa, S. aureus,* and methicillin-resistant *S. aureus,* could grow and colonize on the burn site because of the imbalance between their pathological effects and the efficiency of the human immune system [28,29]. The topical route was considered advantageous for burn healing because it allows the rapid and easy delivery of drugs in a large amount to the burn surface, has better patient compliance, and has a low chance of systemic toxicity [30]. Some well-known antimicrobial drugs such as silver might not be very advantageous for burn treatment owing to their ability to cause cytotoxicity, argyria, or dyschromia. Therefore, some new agents such as CnO and SMV that are reported to have good wound-healing ability could be better and safer alternatives.

### 3.1. Box–Behnken Design Analysis

**Figure 1:**
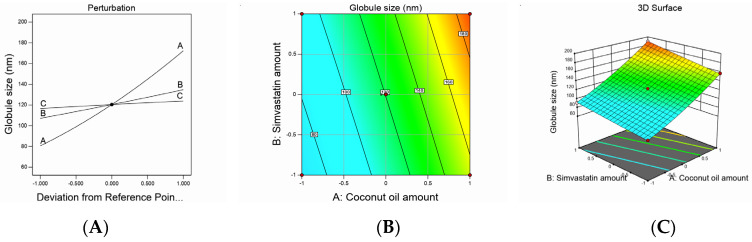
Graphic representation of the effect of independent variables on globule size for different SMV-CnO SNEDDS formulations. (**A**) Main effect plot. (**B**) Contour plot. (**C**) Three-dimensional surface plot.

**Figure 2:**
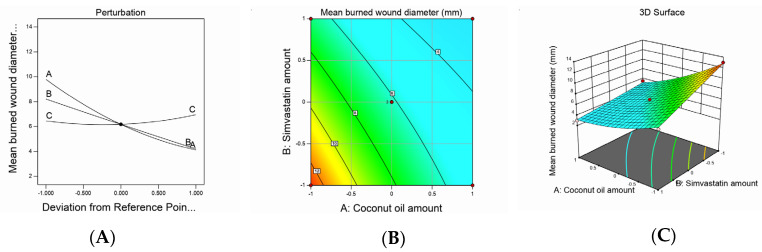
Graphic representation of the effect of independent variables on mean burn wound diameter for different SMV-CnO SNEDDS formulations. (**A**) Main effect plot. (**B**) Contour plot. (**C**) Three-dimensional surface plot.

**Figure 3:**
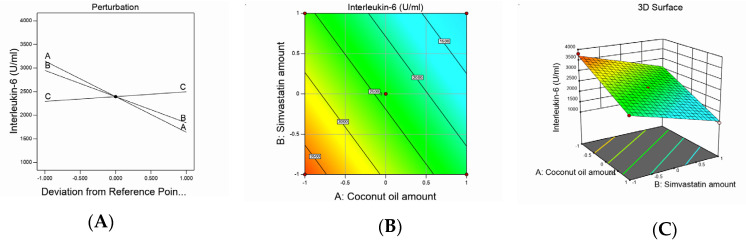
Graphic representation of the effect of independent variables on IL-6 levels for different SMV-CnO SNEDDS formulations. (**A**) Main effect plot. (**B**) Contour plot. (**C**) Three-dimensional surface plot.

**Figure 4:**
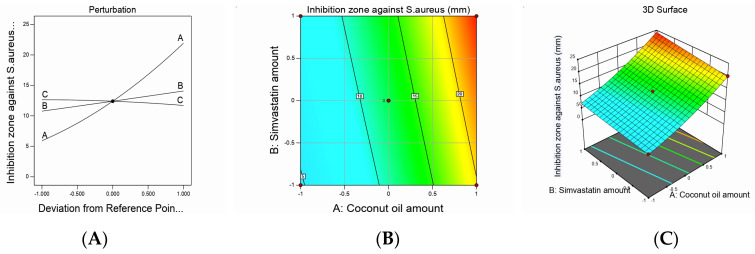
Graphic representation of the effect of independent variables on the inhibition zone against *S. aureus* for different SMV-CnO SNEDDS formulations. (**A**) Main effect plot. (**B**) Contour plot. (**C**) Three-dimensional surface plot.

Design-Expert software (12.0.6.0, Stat-Ease, Inc., Minneapolis, MN, USA) developed the study’s experimental design to evaluate the effect of each independent variable and its interactions on the dependent measured responses, measure the design parameters, and study the model fit and adequacy. The selected model validation was statistically tested using *F*-values at a 95% confidence interval (*p* < 0.05) and the analysis of variance (ANOVA) utilizing Design-Expert software (12.0.6.0, Stat-Ease, Inc., Minneapolis, MN, USA). Moreover, checkpoint analysis was adopted to evaluate the validity and accuracy of the developed mathematical model for dependent response predictions. Surface responses and the overlay plot of the desired responses corresponding to the optimal region where the optimal nanoemulsion can be obtained were produced. Finally, desirability values were assigned, and the optimum parameters were compared for formulation assessment. The effect of the investigated factors on the measured responses was explained by the contour and three-dimensional surface plots, as illustrated in Figure 1, Figure 2, Figure 3 and Figure 4.

### 3.2. Formulation and Characterization of SMV Nanoemulsion

#### Nanoemulsion Droplet Size and Polydispersity Index

The physicochemical character of the nanoemulsions is a key factor to consider before the fabrication process, particularly for a topical delivery of a studied agent [31].

The droplet size is the most important parameter that could differentiate emulsions into microemulsions or nanoemulsions. In the current study, the droplet size of the developed nanoemulsions ranged between 65 and 195 nm, as seen in Table 2, with an acceptable polydispersity index (i.e., ranging from 0.1 to 0.3), which revealed good formulation homogeneity and a satisfactory size distribution.

Droplet size values were subjected to a quadratic model of polynomial analysis. The Box–Behnken design revealed the employed model’s efficiency in investigating the significant impact of the amount of CnO (A), amount of SMV (B), and Smix ratio (C) on the nanoemulsions’ droplet size. The proposed quadratic model exhibited an adjusted R^2^ of 0.9979 and predicted R^2^ of 0.9962, which were in very close accordance, as shown in Table 3. Data analysis by ANOVA gave the following Equation:

*Globule size* = +120.41 + 45.83*A* + 13.78*B* +3.66*C* + 3.00*AB* − 0.2500*AC* + 0.6419*BC* + 6.02*A²* + 0.5203*B²* − 0.2297*C²* (1)

As noticed, all the studied factors exerted a significantly positive effect on the droplet size at a *p*-value of less than 0.0001; hence, increasing the amounts of CnO and SMV and using a higher Smix ratio would end up increasing the droplet size. Increasing the amount of oil could have afforded a larger space for the drug to be accommodated and, hence, resulted in larger droplets. In addition, when the amount of oil was increased, there was a corresponding decrease in the Smix ratio, which led to a decrease in the ability of the used surfactant and cosurfactant to lower the oil droplet size so that finally, larger oil globules were obtained. In the meantime, increasing the amount of SMV also could have resulted in droplet swelling and a yielding of bigger emulsion droplets. Similar results were reported in the literature [32,33].

The decrease in droplet size that accompanied the low level of the Smix ratio could be ascribed to the higher percentage of cosurfactant present in the low Smix ratio. It is known that the cosurfactant used (i.e., Lauroglycol) is a better solubilizer for SMV than the surfactant used (i.e., Cremophor EL), so using higher amounts of Lauroglycol led to a greater ability of the Smix to downsize the oil globules and yield smaller droplets. These results were in agreement with previously reported findings [34]. It was noteworthy that a significantly positive interaction was found between the amounts of CnO and SMV used, whereas the interaction between the SMV amount and Smix ratio employed was insignificantly positive. On the other hand, the interaction between the CnO amount and Smix ratio was insignificantly negative. The studied factors’ effect on droplet size was explained by the contour and three-dimensional surface plots, as illustrated in Figure 1.

### 3.3. Assessment of Wound Healing

#### Mean Burn Wound Diameter Measurement

Wound healing is a complex dynamic process that involves several consecutive and interacting steps, such as inflammation, epithelialization, cell proliferation, and the production of new collagen [35]. In the present research, the burn wound–healing effect of the developed formulations was investigated and the wound diameter was measured to confirm wound healing. The burn wound diameter values exhibited a quadratic model of polynomial equations, and the statistical design adopted used the model efficiency to detect the effect of the studied factors on the diameter of the burn wounds. The suggested model attained an adjusted R^2^ value of 0.9890, which was in very close agreement with a predicted R^2^ value of 0.9689, as shown in Table 3. The equation below was obtained based on the ANOVA statistical analysis:

*Mean burned wound diameter* = +6.18 − 2.83*A* − 2.00*B* + 0.2500*C* + 1.00*AB* − 0.2500*AC* − 0.2635*BC* + 0.7838*A²* + 0.0338*B²* + 0.5338*C²* (2)

The mean burn wound diameter of the animals treated with a nanoemulsion ranged from 3 to 12.5 mm, as seen in Table 2. It was observed that the CnO and SMV amounts had a significantly negative effect on the mean burn wound diameter values, whereas the Smix ratio exerted a significantly positive effect on the same parameter at a *p*-value of less than 0.0001. The ability of the SMV to decrease the burn wound diameter may be ascribed to its ability to enhance vascular endothelial growth factor production at the wound site, which is critical for new blood vessel growth [17]. In addition, SMV was also believed to decrease mevalonate and farnesyl pyrophosphate production, resulting in increased epithelialization and revival of injured skin. Furthermore, CnO could have decreased the burn wound diameter owing to its ability to enhance fibroblast proliferation and keratinocyte migration. Similar results were seen in the literature [36].

The increase in the burn wound diameter observed with the increase in the Smix ratio could be understood based on the droplet size results previously explained. As mentioned earlier, the increase in the Smix ratio produced larger emulsion droplets, which might have led to a decrease in the globule surface area and, hence, a decreased nanoemulsion ability to release SMV and decrease the amount of drug available at the injury site.

Noticeably, the interaction between the SMV (B) and CnO (A) amounts was significantly positive (*p* < 0.0001), whereas the interaction between the SMV amount (B) and Smix ratio (C) and between the CnO amount (A) and Smix ratio (C) was insignificantly negative, at *p*-values of less than 0.0754 and less than 0.0647, respectively. The impact of the studied factors on the mean burn wound diameter is illustrated by contour and three-dimensional surface plots, as presented in Figure 2.

### 3.4. Interleukin-6 Measurements

IL-6 is an abundantly produced cell protein that helps in regulating immune system responses. Classically, the IL-6 level becomes elevated in response to microbial infection, inflammation, immune system diseases, and (sometimes) cancer, so it can be considered a useful marker of inflammation and immune system activation [37].

The obtained mean values of the IL-6 serum levels attained a linear model of polynomial equations based on the adopted statistical design, which evaluated the impact of the studied independent variables on IL-6 serum levels. The model obtained showed an adjusted R^2^ value of 0.9822, which was very close to the predicted R^2^ value of 0.971, as seen in Table 3. ANOVA analysis of the obtained data yielded the following equation:

*Interleukin-6* = +2393.68 − 754.17*A* − 554.69*B* + 101.56*C* (3)

The measured IL-6 serum levels varied between 1000 and 3800 U/mL in the studied animal groups, as shown in Table 2. It was observed that the SMV and CnO amounts had a significantly negative effect on IL-6 levels, whereas the Smix ratio had a significantly positive effect on the same parameter (*p* < 0.0001). The decrease in IL-6 levels observed with an increase in the SMV amount could be referred to as an SMV antiinflammatory characteristic [38]. It is well established that statins can lower the production of proinflammatory cytokines [39] by inhibiting HMG-CoA reductase, which would activate the mevalonate pathway. Consequently, statins could reduce isoprenylated and geranylgeranylated protein production and, especially, Ras protein prenylation. The inhibition of Ras proteins decreases the activity of transcription factor nuclear factor κappa B (NF-κB), which is a key element in several inflammatory reactions [40]. These facts explain the IL-6-lowering effect of SMV.

It was also observed that the increase in the CnO amount decreased IL-6 levels. These results might be explained in the light of CnO composition. CnO is rich in polyphenols, which are well reported to affect inflammatory chain reactions so that pathogenesis is avoided and healthy effects are enforced. CnO polyphenols initiate an indirect mechanism to release antiinflammatory signaling molecules and counteract inflammation [41,42].

The direct relationship between the Smix ratio and the IL-6 serum level might be attributed to the larger globule size observed with higher Smix ratios and, hence, the smaller surface area afforded for drug release and permeation, leading to a decreased efficiency of the studied agents to decrease IL-6 levels. The effect of the independent variables on IL-6 serum levels was demonstrated by contour and three-dimensional surface plots, as presented in Figure 3.

### 3.5. Antimicrobial Activity of the Developed Nanoemulsions

Antimicrobial activity of the developed nanoemulsions was examined against *S. aureus* by measuring the inhibition zone in plates treated by the nanoemulsion formulations. As shown in Table 2, the diameter of the inhibition zone of *S. aureus* fluctuated between 4 and 24 mm.

The inhibition zones against *S. aureus* acquired a quadratic model of polynomial equations based on the adopted statistical design, which evaluated the influence of the studied factors on the measured diameters of inhibition zones. The model obtained exhibited an adjusted R^2^ value of 0.9951, which was in accordance with a predicted R^2^ value of 0.9873, as presented in Table 3. ANOVA analysis of the obtained data yielded the following equation:

*Inhibition zone against S. aureus*= +12.41 + 8.00*A* + 1.66*B* − 0.4687*C* + 0.5000*AB* − 0.2500*AC* − 0.3581*BC* + 1.52*A²* + 0.0203*B²* − 0.2297*C²* (4)

As noticed, the SMV and CnO amounts had a significantly positive effect on the inhibition zones, whereas the Smix ratio had a significantly negative effect on the same parameter (*p* < 0.0001).

The increase in the SMV amount increased the diameter of the bacterial growth inhibition zone owing to its antimicrobial activity; this could be a result of its pleiotropic characters because it was reported that SMV could suppress *S. aureus* cell growth and enhance apoptosis [43]. It was also hypothesized that the antibacterial activity of statins could be due to the interaction of the methyl group from the gem-dimethyl moiety or cyclopropyl ring present in SMV with the alanine residues of Gram-positive bacteria via hydrogen bonding or the van der Waals forces [44]. That interaction may cause structural deformation of bacteria [45] or a shortage in available alanine residues, hence reducing bacterial biofilm formation and the adherence of a biofilm to the skin surface.

In addition, the antibacterial effect of CnO, which is obvious in the results of the *S. aureus* inhibition zone test, might be a result of its rich content of diglycerides and FFAs. A combination of the FFA, lauric acid, and monoglycerides such as monolaurin is reported to have a good antibacterial effect against a wide range of bacterial strains [9].

The Smix ratio exhibited a significantly negative effect on the inhibition zone against *S. aureus* (*p* < 0.0096). Such results could be explained by virtue of the droplet size. When the Smix ratio was increased, the droplet size also increased. This meant that a smaller surface area would be available for drug release and penetration through the bacterial cell membrane, leading to decreased bacterial death and, ultimately, a decreased *S. aureus* inhibition zone.

The statistical analysis results revealed the presence of a significantly positive interaction between SMV (B) and CnO (A) amounts (*p* < 0.02); the interaction between the SMV amount (B) and Smix ratio (C) and between the CnO amount (A) and Smix ratio (C) was insignificantly negative at *p*-values of less than 0.0952 and less than 0.2227, respectively. The studied factors’ effects on the inhibition zone against *S. aureus* are illustrated by contour and three-dimensional surface plots, as presented in Figure 4.

### 3.6. Optimization of Nanoemulsion Formulations

**Figure 5:**
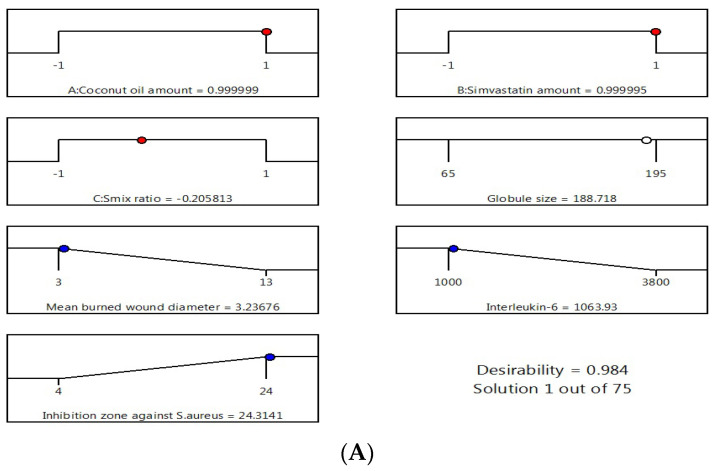

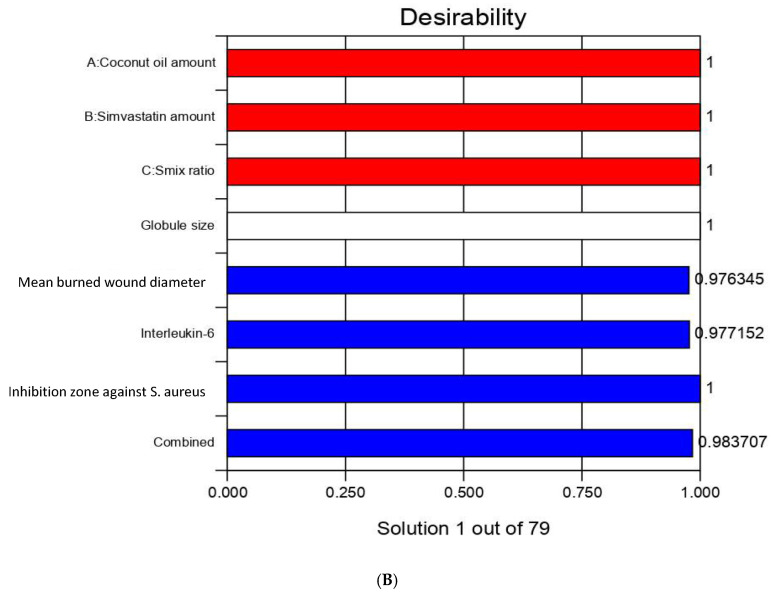
Desirability ramp and bar chart for optimization. (**A**) Desirability ramp shows the levels for independent variables and predicted values for the responses of the optimum formulation. (**B**) Bar chart shows the desirability values for the combined responses.

After performing all the previous tests, the optimum nanoemulsion formulation was identified with the most suitable characteristics. The employed Design-Expert software proposed several solutions for different combinations of the studied factors’ levels. The optimum formulation was composed of 225 mg of CnO and 20 mg of SMV and had a Smix ratio of 1.8:1. The obtained optimum formulation acquired a droplet size of 186 nm, ZP value of 20 ± 1.2 mV, and PDI of 0.2 ± 0.03 indicating reasonable stability of the optimal formula, a mean burn wound diameter of 3 mm, an IL-6 value of 1045 U/mL, and an inhibition zone against *S. aureus* of 24 mm with 0.9840 desirability (Figure 5 illustrates the desirability plot). Table 4 reveals that the predicted and actual values of the optimum nanoemulsion formulation characteristics were very close, with no major differences (*p* > 0.05), and this verified the equations’ validity and predictability.

### 3.7. Checkpoint Analysis

**Figure 6:**
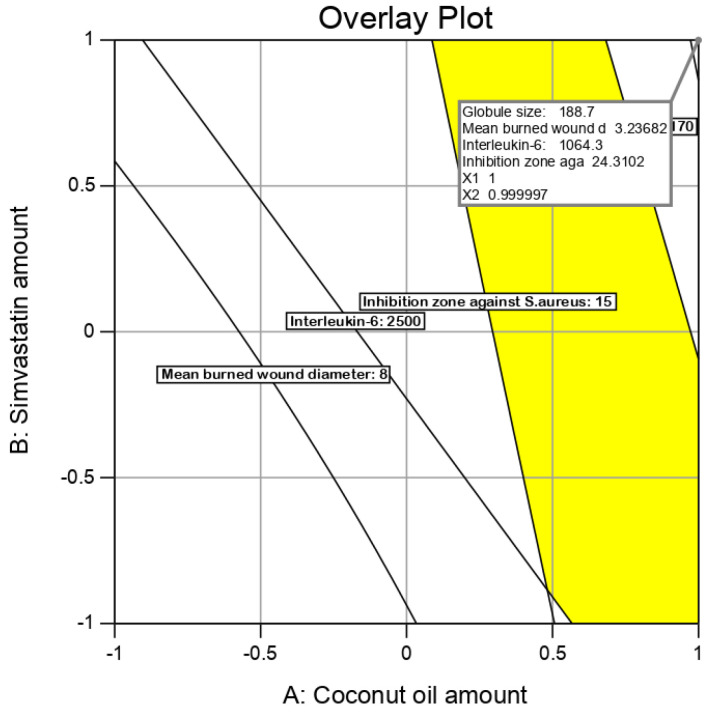
Overlay plot for the optimal SMV-CnO SNEDDS region determination.

Values of the expected and adjusted R^2^ of the studied responses were in close agreement, proving the significance and predictive capacity of the design. Moreover, the experimental/predicted ratios with a percentage error of less than 10% and acceptable residuals were found between the real and predicted responses, showing a lack of curvature in the responses and the validity of the model (Table 5). Figure 6 shows the overlay plot for the optimal region.

### 3.8. Characterization and Evaluation of Optimum Nanoemulsion Formulation

The optimum formulation suggested by the software was developed, characterized, and compared against four different formulations, the components of which are seen in Table 6. As can be seen in Table 6, the optimized formulation decreased the mean burn wound diameter by 5.3-fold compared with normal saline and by 4.6-fold compared with the SMV aqueous dispersion. This result indicated the usefulness of SMV and coconut oil in the healing of wounds. The mean burn wound diameter was reduced by 2.3-fold and 2.6-fold compared with F2 and F1, which contained SMV alone, and CnO alone, respectively. This indicated a synergistic effect for the combining of SMV and coconut oil in the same formulation.

Moreover, the optimum formulation lowered the IL-6 levels by 4.25-fold and 3.39-fold compared with normal saline and the SMV aqueous dispersion, respectively. Such results proved the superior antiinflammatory properties of SMV and coconut oil employed in the optimum formulation. In addition, the IL-6 level obtained after treatment with the optimized formulation was decreased by 2.33-fold compared with F1, which contained no SMV, and by 2.67-fold compared with F2, which contained oleic acid rather than coconut oil. These findings show the preferred synergistic effect of the SMV and coconut oil combination in wound treatment.

**Figure 7:**
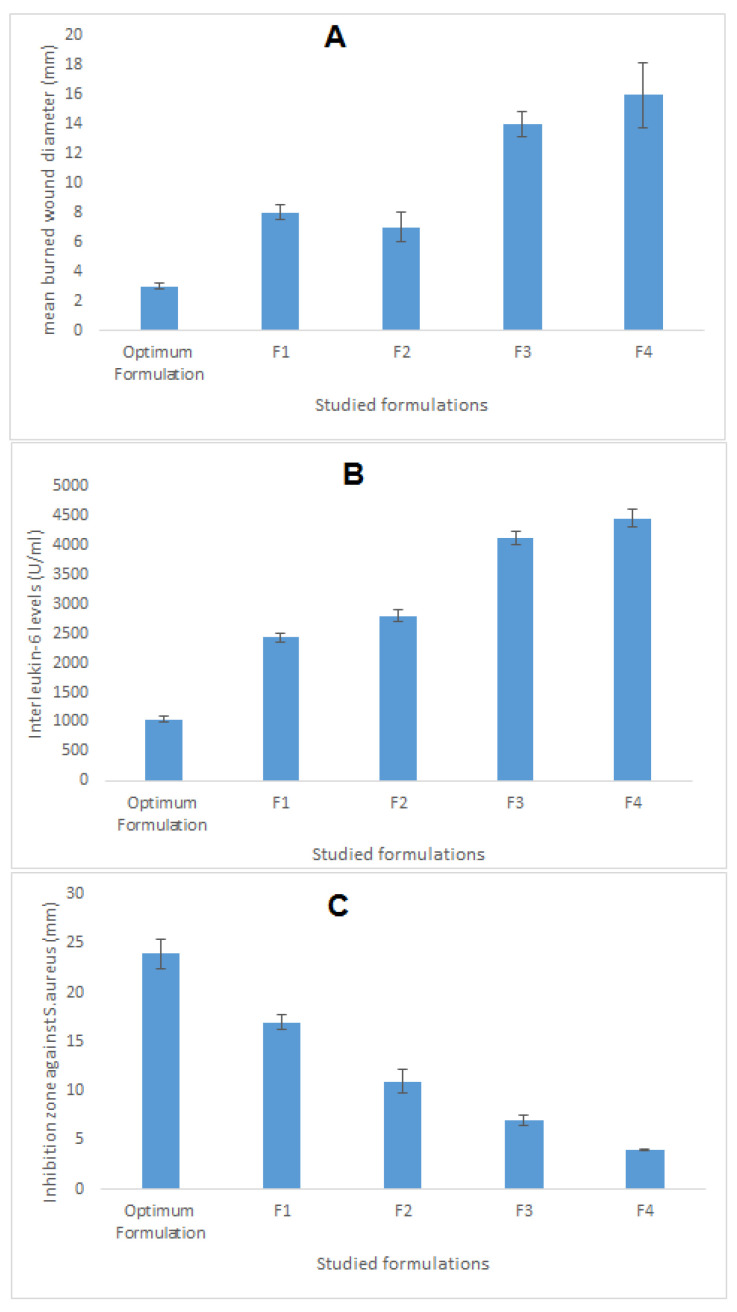
Wound-healing efficacy of the optimized SMV-CnO SNEDDS formulation compared with the same parameters in four different controls. (**A**) Mean burn wound diameter. (**B**) IL-6 level. (**C**) Inhibition zone against *S. aureus*.

Values of the inhibition zone against *S. aureus* obtained after treatment with the optimized formulation were also assessed and compared with the mentioned control groups. Briefly, the optimum formulation increased the inhibition zone diameter by 3.42-fold and 6-fold compared with the SMV aqueous dispersion and normal saline, respectively, illustrating the potential antimicrobial activity of the studied SMV and coconut oil. Furthermore, the inhibition zone diameter obtained after treatment with the optimized formulation was enhanced by 1.41 = fold compared with F1, which contained no SMV, and by 2.18-fold compared with F2, which contained oleic acid rather than coconut oil. These data reveal the additive effect of SMV and coconut oil and the ability to use them in combination as a therapy in burn wound management. Figure 7 illustrates the wound-healing efficacy of the developed optimum formulation against the studied control groups.

## 4. Conclusions

In the current study, the Box–Behnken design was employed for the optimization of a coconut oil–based nanoemulsion loaded with SMV that was intended for the topical treatment of burn wounds. The developed nanoemulsions acquired an adequate droplet size of between 65 and 195 nm, indicating the formation of nanoemulsions. The statistical design proved the important synergistic effect of coconut oil and SMV for burn wound management. This combination potentiated the wound closure ability, anti-inflammatory properties, and antimicrobial effects of each of the components. The optimum formulation achieved up to a 5.3-fold decrease in the mean burn wound diameter, a 4.25-fold decrease in the IL-6 level, and a 6-fold increase in the inhibition zone against *S. aureus* when compared with different control formulations. Therefore, the designed nanoemulsions containing a combination of coconut oil and SMV could be considered promising platforms for the treatment of chronic and burn wounds.

## Figures and Tables

**Table 1:** Independent variables and their levels, along with dependent variables and their constraints, in the Box–Behnken design of nanoemulsion formulations.

Independent Variables	Levels		
	−1	0	1
A = Coconut oil amount (mg)	125	175	225
B = Simvastatin amount (mg)	5	10	20
C = S mix ratio (surfactant: cosurfactant)	1:1	1:2	1:3
Dependent variables	Constrains		
Y_1_ = Droplet size (nm)	Minimize		
Y_2_ = mean burned wound diameter (mm)	Minimize		
Y_3_ = Interleukin-6 (U/mL)	Minimize		
Y_4_ = inhibition zone against *S. aureus* (mm)	Maximize		

**Table 2:** Box–Behnken design and responses of SMV-loaded self-nanoemulsion drug delivery systems (SNEDDSs).

	A	B	C	Y_1_	Y_2_	Y_3_	Y_4_
Run	Coconut Oil Amount	Simvastatin Amount	Smix Ratio	Globule Size (nm)	Mean Burned Wound Diameter (mm)	Interleukin-6 (U/mL)	Inhibition Zone against *S. aureus* (mm)
1	1	0	1	174	4.5	1750	21
2	−1	1	0	91	6.5	2450	7
3	0	1	1	138	5	2000	13
4	1	−1	0	157	5.5	2200	20
5	0	0	0	120	6	2250	13
6	−1	0	−1	78	10	3150	6
7	0	0	0	118	6	2280	12
8	1	0	−1	169	4.5	1600	22
9	0	1	−1	130	5	1850	15
10	−1	−1	0	71	13	3800	5
11	−1	0	1	84	11	3400	6
12	0	−1	−1	105	8	2850	11
13	0	0	0	123	6.5	2400	12
14	1	1	0	189	3	1000	24
15	0	−1	1	110	9	3000	10
16	−1	−1	−1	65	12.5	3550	4
17	1	−1	−1	152	5.5	2050	20
18	−1	1	1	97	7.5	2600	6
19	1	1	1	195	3.5	1300	23

**Table 3:** Regression analysis results for all responses.

Dependent Variables	R2	Adjusted R2	Predicted R2	*p*-Value	*F*-Value	Adequate Precision
Y_1_	0.9990	0.9979	0.9962	0.0001	962.96	108.41
Y_2_	0.9945	0.9890	0.9689	0.0001	181.19	4.73
Y_3_	0.9852	0.9822	0.9761	0.0001	332.59	2.477 × 10^5^
Y_4_	0.9976	0.9951	0.9873	0.0003	411.01	10.38

**Table 4:** Actual and experimental values of the optimized nanoemulsion formulation (means ± SD, *n* = 3).

Solution	Coconut oil Amount (mg)	SMV Amount (mg)	Smix Ratio	Droplet Size (nm)	Mean Burned Wound Diameter (mm)	Interleukin-6 (U/mL)	Inhibition Zone against *S. aureus* (mm)	Desirability
Predicated value	225	20	1.8:1	188.7	3.2	1063.9	24.3	0.9840
Experimental value	225	20	1.8:1	186.0 ± 2.5	3.0 ± 0.10	1045.0 ± 15	24.0 ± 0.5	0.9840

**Table 5:** Composition and actual and predicted responses of the optimal nanoemulsion formulation.

Factor	Optimal Value	Response Variable	Actual Value	Predicted Value	% Prediction Error ^a^
A: Coconut oil amount (mg)	225	Droplet size (nm)	186	188.7	−1.45
B: SMV amount (mg)	20	Mean burned wound diameter (mm)	3	3.2	−6.66
C: Smix ratio	1.8:1	Interleukin-6 (U/mL)	1045	1063.9	−1.72
		Inhibition zone against *S. aureus* (mm)	24	24.3	−1.25

^a^: negative sign indicated that predicted value higher than actual value.

**Table 6:** Characterization of optimum formulation in comparison with control groups (means ± SD, *n* = 3).

Run	A: CnO Amount	B: SMV Amount	C: Smix Ratio	Globule Size	Mean Burned Wound Diameter	Interleukin-6	Inhibition Zone against *S. aureus*
				(nm)	(mm)	(U/mL)	(mm)
Optimum formulation	225 mg	20	1.8:1	186	3 ± 0.2	1045 ± 55	24 ± 1.5
F1	225 mg	0	1.8:1	144	8 ± 0.5	2435 ± 76	17 ± 0.8
F2	Oleic acid	20	1.8:1	198	7 ± 1.0	2800 ± 95	11 ± 1.2
SMV aqueous dispersion	0	20	0	650	14 ± 0.9	4115 ± 120	7 ± 0.5
Normal Saline	0	0	0	-	16 ± 2.2	4450 ± 150	4 ± 0.1
